# Evaluating the diversity of Neotropical anurans using DNA barcodes

**DOI:** 10.3897/zookeys.637.8637

**Published:** 2016-12-02

**Authors:** Ruth A. Estupiñán, Stephen F. Ferrari, Evonnildo C. Gonçalves, Maria Silvanira R. Barbosa, Marcelo Vallinoto, Maria Paula C. Schneider

**Affiliations:** 1 Instituto Federal da Paraíba, Campus Cabedelo, Cabedelo, Brazil; 2 Universidade Federal de Sergipe, São Cristóvão, Brazil; 3 Universidade Federal do Pará, Belém, Brazil; 4 Universidade Federal do Pará, Campus de Bragança, Bragança. Brazil; 5 Universidade Federal do Pará, Belém. Brazil

**Keywords:** Amazon basin, amphibians, COI, DNA barcoding, identification, taxonomy

## Abstract

This study tested the effectiveness of COI barcodes for the discrimination of anuran species from the Amazon basin and other Neotropical regions. Barcodes were determined for a total of 59 species, with a further 58 species being included from GenBank. In most cases, distinguishing species using the barcodes was straightforward. Each species had a distinct COI barcode or codes, with intraspecific distances ranging from 0% to 9.9%. However, relatively high intraspecific divergence (11.4–19.4%) was observed in some species, such as *Ranitomeya ventrimaculata*, *Craugastor fitzingeri*, *Hypsiboas leptolineatus*, *Scinax fuscomarginatus* and *Leptodactylus knudseni*, which may reflect errors of identification or the presence of a species complex. Intraspecific distances recorded in species for which samples were obtained from GenBank (*Engystomops pustulosus*, *Atelopus varius*, *Craugastor podiciferus*, and *Dendropsophus labialis*) were greater than those between many pairs of species. Interspecific distances ranged between 11–39%. Overall, the clear differences observed between most intra- and inter-specific distances indicate that the COI barcode is an effective tool for the identification of Neotropical species in most of the cases analyzed in the present study.

## Introduction

Many amphibian groups are morphologically homogeneous and tend to lack clear diagnostic traits. This means that, while there have been a number of recent advances, the taxonomy of amphibians is poorly resolved in general (see e.g. Darst and Cannatella 2004; Faivovich et al. 2005; Frost et al. 2010; Grant et al. 2006; Roelants et al. 2007; Vences et al. 2003). In particular, the intrageneric diversity of the amphibians appears to be underestimated in most cases (e.g., Bossuyt et al. 2004; Crawford et al. 2010; De la Riva et al. 2000; Fouquet et al. 2007; Vieites et al. 2009). In this context, the accelerating global decline and changes in amphibian populations (Hoffmann, et al. 2010, McCallum 2007; Stuart et al. 2004; Narins et al. 2014), as well as the cryptic diversity reported for several taxa (Fouquet et al. 2007; Crawford et al. 2013), implies that many still undescribed species may be disappearing from the Neotropical region before they have even been identified (Collins 2010).

The increasing availability of molecular data has reinforced the conclusion that morphological evolution in amphibians is often cryptic, resulting in a revitalization of amphibian taxonomy (e.g. Real et al. 2005; Vieites et al. 2009; Rowley et al. 2010; Stuart et al. 2006; Funk et al. 2012; Xia et al. 2012; Crawford et al. 2013). Rapidly-evolving genes may overwrite the evidence of ancient affinities, but are extremely useful for the understanding of recent divergence among closely-related species. Mitochondrial DNA (mtDNA) has been widely used in phylogenetic studies of animals because it evolves much more rapidly than nuclear DNA, resulting in the accumulation of differences between closely-related species (Brown et al. 1979; Moore 1995; Mindell et al. 1997). The taxonomic reviews at the species level now almost always include some form of analysis of mtDNA divergence. A number of species of the genus *Rana* have been recognized in recent years, based on molecular methods (Newman et al. 2012), for example, and through comparisons with other amphibian species (Channing et al. 2013; Hasan et al. 2014; Biju et al. 2014).

Short DNA sequences from a standardized region of the genome can provide a DNA “barcode” for the identification of species (Hebert et al. 2003), and may provide a substitute for more traditional molecular approaches, which have been used for the identification of amphibian taxa for some time (Larson and Chippindale 1993). A 648-bp region of the mitochondrial Cytochrome Oxidase I (COI) gene is commonly used as a barcode for the identification of animal species, given that it is easily sequenced and provides excellent resolution for the identification of taxa, especially when combined with the analysis of other traits (Pereyra et al. 2016). This is supported by the considerable divergence in sequences found by Hebert et al. (2003) between 13,000 pairs of closely-related animal species, and reinforces the need for the analysis of more than a single, short sequence of DNA, which may produce inconclusive results (Blotto et al. 2012, Pereyra et al. 2016).

The usefulness of COI as a DNA barcode has been evaluated in Malagasy mantellids and North American plethodontid salamanders (Vences et al. 2005a), Holarctic amphibians (Smith et al. 2008), and Asiatic salamanders of the family Hynobiidae (Xia et al. 2012). In the Neotropical zone, COI has been tested in amphibians from Panama and the Guianan Shield (Crawford et al. 2010, 2013; Hawkins et al. 2007). Variations in the performance of COI as a DNA barcode have provoked doubts on the effectiveness of the approach for the identification of species (Vences et al. 2005b). The main limitation on the use of COI in amphibians is the lack of a universal primer for the PCR-mediated amplification of the DNA of different species (Vences et al. 2012). In many cases, the overlap found between intraspecific and interspecific distances reduces the reliability of species identification (Vences et al. 2005a; Hawkins et al. 2007). Given this, Vences et al. (2005b) recommended the use of 16S rRNA as a DNA barcode, rather than COI.

Using a combination of primers, COI sequences were used to successfully identify 94% of Holarctic amphibians, and showed that the overlap between intra- and inter-specific distances was the result of hybridization, the presence of species complexes or taxonomic problems (Smith et al. 2008). In many cases, there was no overlap in these distances. Overall, then, the COI barcode presented the same problems encountered in the analysis of any other group of animals (Smith et al. 2008; Crawford et al. 2010; Hawkins et al. 2007; Vences et al. 2012).

In this context, the present study evaluated the potential of the mitochondrial COI gene as a barcode, used in combination with other traits, for the identification of Neotropical amphibians from the Amazon basin and other regions of South America. In particular, the study compares the molecular classification of the specimens with the traditional taxonomy of the group.

## Material and methods

### Study area and samples

**Figure 1.**
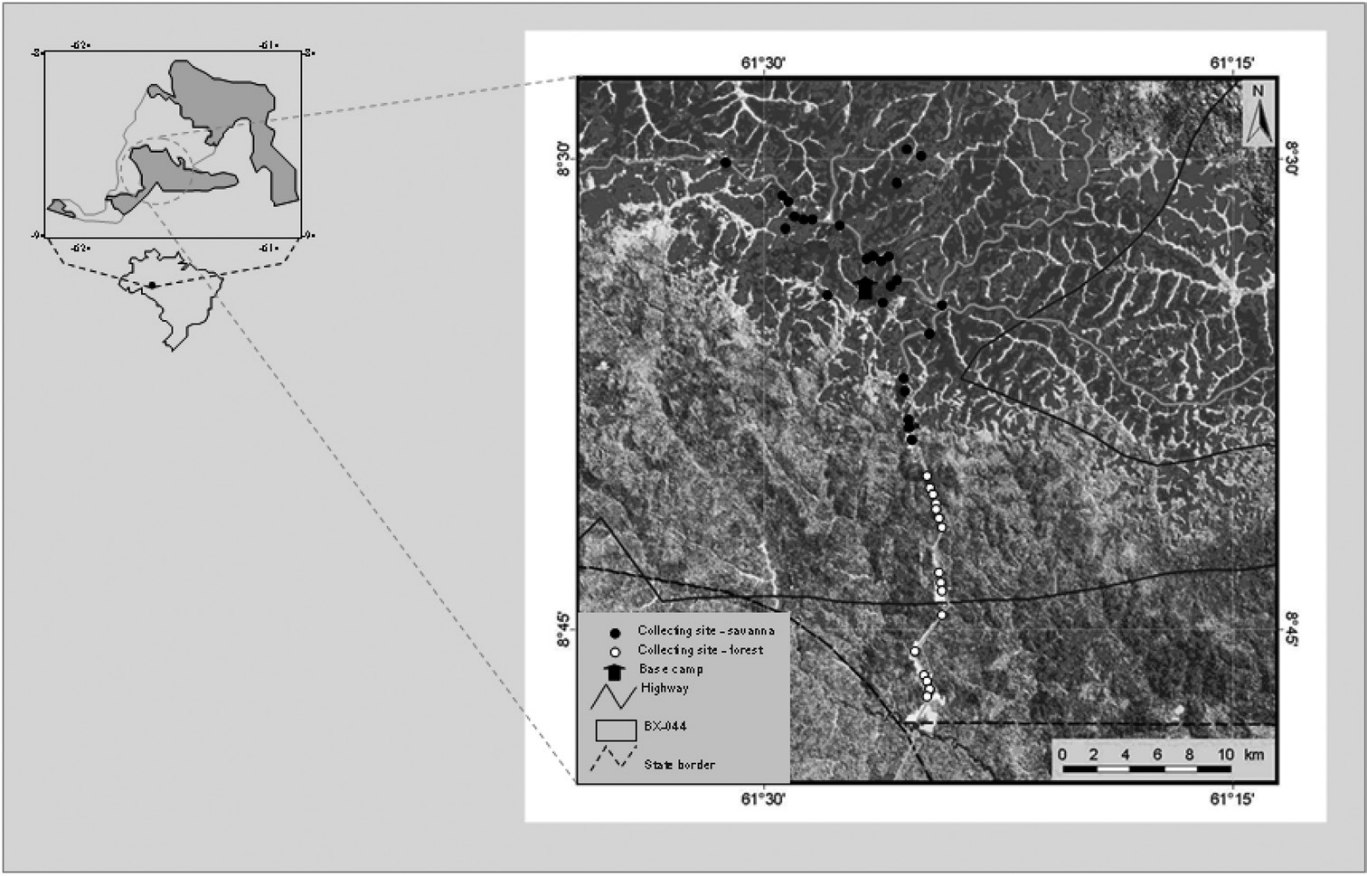
The BX044 priority area for conservation showing the sites at which anuran specimens were collected.

In order to establish a reference site for the evaluation of a barcoding approach for Amazonian vertebrates, a field survey was conducted in the BX044 polygon in the southwestern Amazon basin, an area considered to be of the highest importance for the conservation of the biome’s biological diversity (Pronabio, 2002). The polygon covers an area of 5270 km^2^ and is located between latitudes 08°02'52" and 08°54'46" S, and longitudes 60°50'24" and 62°10'13"W, within the Madeira-Tapajós interfluve (Fig. 1). This interfluve is poorly studied and has few few protected areas, with no more than six percent of its total area located within conservation units of any kind (Ferreira et al. 2001). Notwithstanding, it encompasses a unique complex of habitats including open forests, savanna, forest-savanna transition, and gallery forests (Pereira et al. 2004). This mosaic of habitats reflects the position of the study area within the ecotone marking the transition between the Amazonian Hylea and the Cerrado savannas of central Brazil (Nascimento et al. 1988; Stotz et al. 1997).

Specimens were collected in January, 2004, at 74 sites located along the Maderinha, Roosevelt, and Jatuarana rivers, and their tributaries. Specimens were collected in open and dense savanna habitats, gallery and flooded forests, rainforest, and ricefields. The specimens were euthanized with a lethal dose of lidocaine (Brasil 1979). A total of 76 specimens representing 33 species was collected, and 37 sequences were obtained from 17 species, which represent one third of the total number of species analyzed in the present study. The sample was augmented by tissue samples (41 specimens representing 37 species) obtained from other institutions in Brazil and other countries. In addition to these samples, the COI sequences of a number of other amphibian species (see Suppl. material 1) with large sample sizes were obtained from GenBank, to provide a better visualization of the variation in the COI gene in these organisms.

### Specimen identification

Following the extraction of tissue samples, the specimens collected during the present study were preserved for identification at the Goeldi Museum in Belém, Brazil, where they were confirmed by M.S.H. Hoogmoed. The accuracy of COI as a barcode for the identification of species was assessed based on the most recent classification of the amphibians (Frost 2016).

### Molecular methods

Total DNA was extracted from either muscle or liver tissue by the SDS-proteinase K/phenol-chloroform extraction method (Sambrook and Russell 2001). A partial 680-bp fragment of the COI gene was amplified using the 5-CCTGCAGGAGGAGGAGAYCC-3´ and 5-AGTATAAGCGTCTGGGTAGTC-3´ primers (Palumbi 1996). The 25 µL polymerase chain reaction (PCR) mixture contained 0.4-1.2 µL of the DNA template, 2.5 µL 10XPCR buffer, 0.5 µL of each primer (10 pM/µL), 0.6-2.0 µL of MgCl_2_, 1µL dNTPs, and 0.15 µL of *Taq* DNA polymerase. The PCR conditions consistedof 3 min at 94 °C, followed by 35 (or 34) cycles of 50 sec at 94 °C, 50 sec at 55 °C (or 57 and 60 °C), 50 sec at 72 °C and a final extension at 72 °C for 5 min. The DNA was sequenced in both directions using the primers described above in a MegaBace (GE Healthcare) automatic DNA sequencer, using the DYEnamic ET Dye Terminator kit (GE Healthcare).

The sequences obtained were aligned and edited by BIOEDIT v. 7.0.5.3 (Hall 1999). The possible saturation of bases was assessed using a graphic representation of transitions and transversions (Ti-Tv) plotted against Kimura 2 parameters’ distance (Kimura 1980). This analysis was run in DAMBE v. 5.3.105 (Xia 2013).

Pairwise comparisons of COI sequences were conducted for three categories: (i) individuals of the same species, (ii) individuals of the same genus (excluding those of the same species), and (iii) individuals of the same family (excluding those of the same genus). The frequency distribution of intra- and interspecific genetic distances was calculated using MEGA 5 (Tamura et al. 2011), as was a neighbor-joining (NJ) tree based on the K2P model (Kimura 1980). The robustness of the nodes of this tree was estimated by a bootstrap analysis, with 1000 pseudo-replications.

The variability of the COI gene between populations of the same species was also tested using the K2P model, for which the species were selected based on the largest possible sample size (number of specimens) in GenBank (*Atelopus varius*, *Craugastor podiciferus*, *Dendropsophus labialis* and *Engystomops pustulosus*) and the availability of accurate information on their geographic origin. Additional species were included in this analysis (see Suppl. material 1).

## Results

**Figure 2.**
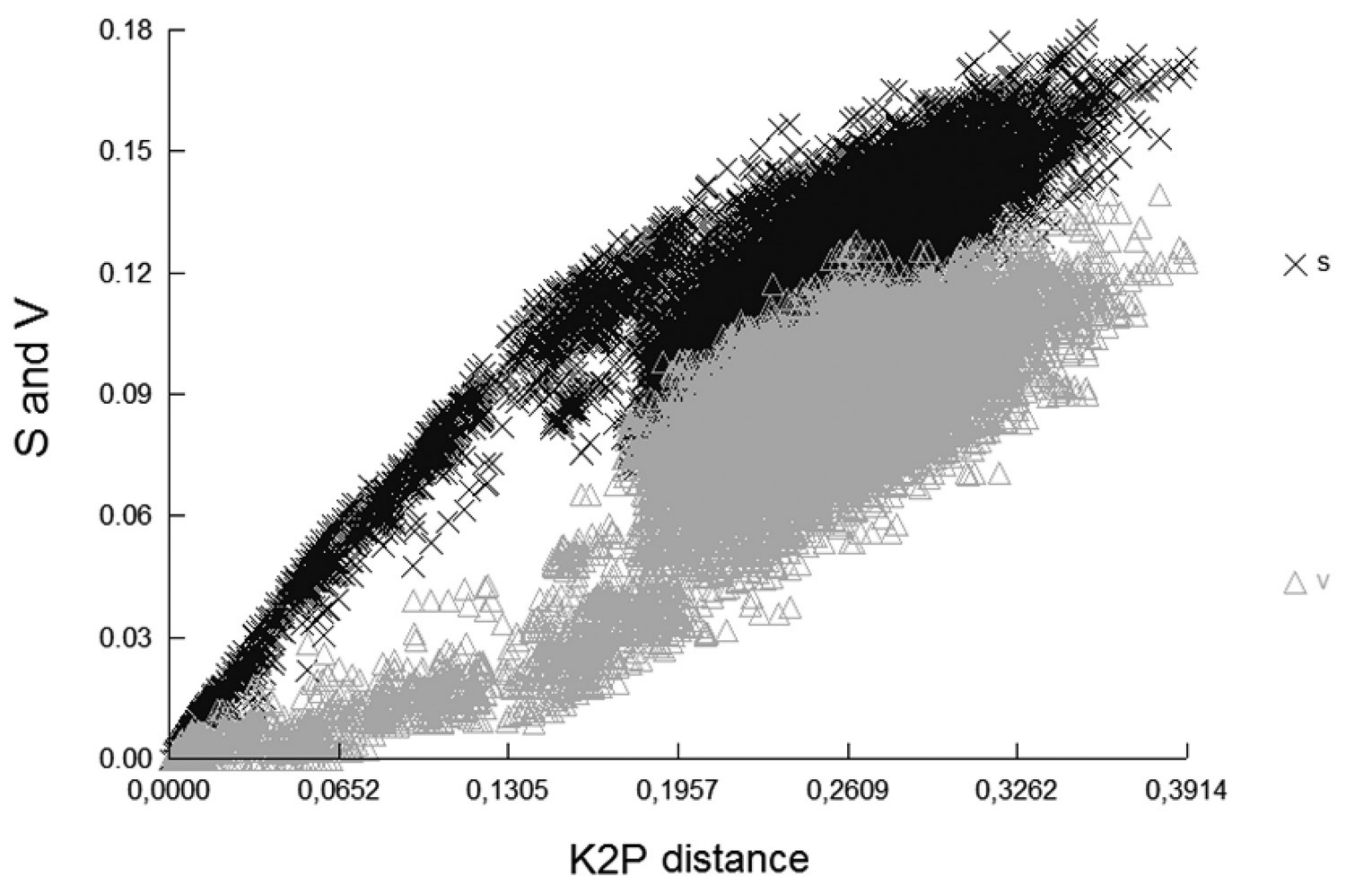
Transition (s) and transversions (v) plotted against the sequence divergence (Kimura 2-parameter distances) for the analyzed anurans.

COI sequences were recovered from 75% (83/110) of the specimens analyzed. Full-length PCR products (640 bps) were amplified from all of these specimens (see Suppl. material 1). Of the 111 species analyzed, sequences of 56 were obtained during the present study and 58 from GenBank (sequences of *Dendropsophus minutus*, *Rhinella marina*, and *Osteocephalus taurinus* were obtained from both sources). Altogether, 410 sequences were analyzed, of which, 78 were obtained in the present study and 332 from GenBank. No evidence of base saturation was found whatsoever (Fig. 2).

### Species identification

**Figure 3.**
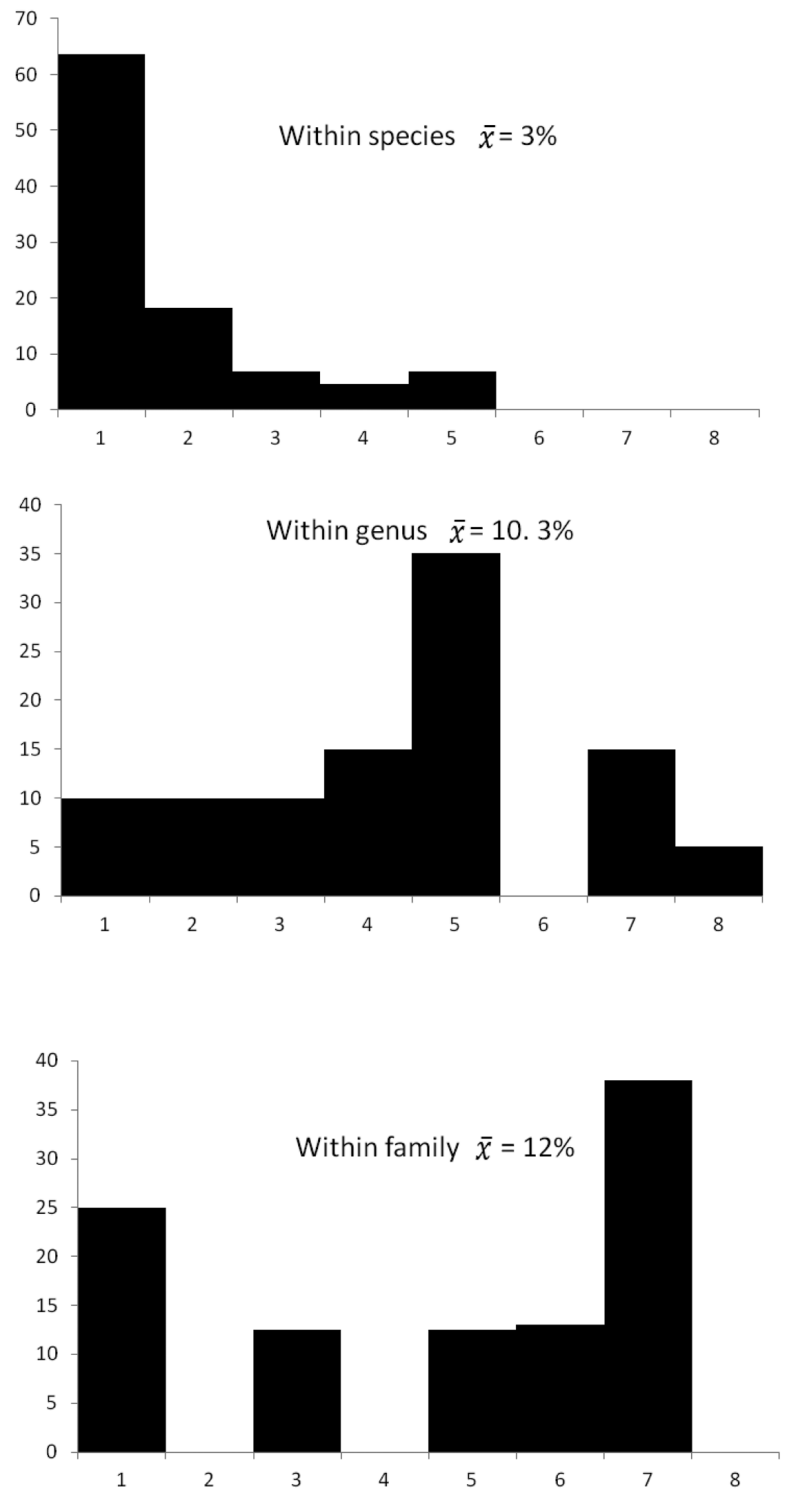
COI sequence divergence (K2P) at various levels of the taxonomic hierarchy for anurans.

The COI barcode identified correctly the species of 94% of the specimens examined (93 of 109 species). The COI sequences obtained for the 36 species represented by two or more specimens were most similar to one another than to those of any other species. In addition, with a few notable exceptions, which are discussed below, the differences in COI sequences between closely-related species were higher than those within species. The mean K2P distance within species was 3.0% (Fig. 3), whereas that between species was 10.3%.

**Figure 4.**
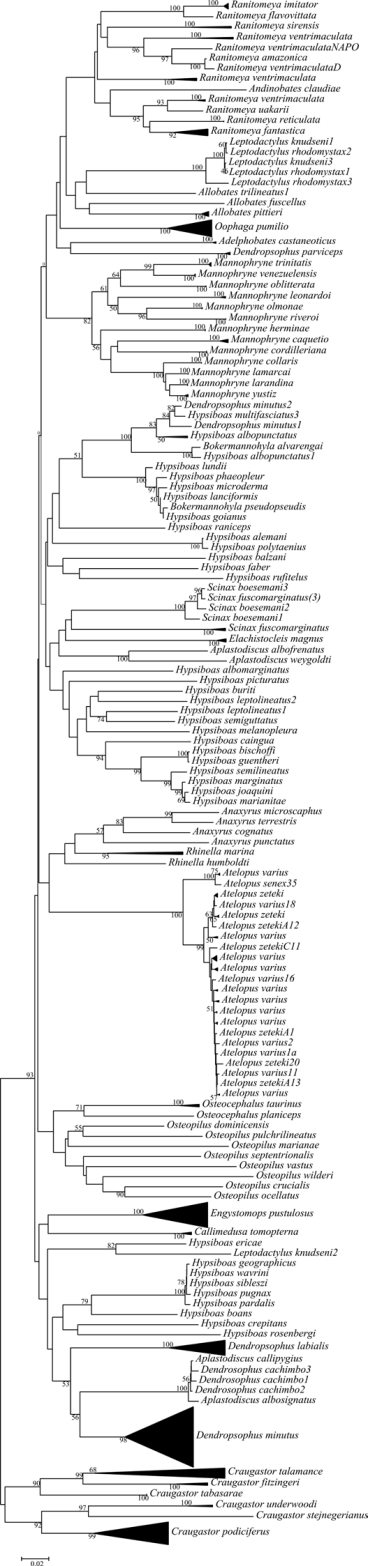
Neighbor-joining (NJ) tree derived from the analysis of COI sequences. The numbers at the nodes represent the percentage bootstrap values.

In most cases, the neighbor-joining (NJ) tree reflected a relatively reduced differentiation within species in comparison with between-species divergence (Fig. 4). Most of the terminal groups include specimens of the same species or genus with bootstrap values of over 85, except for *Ranitomeya*, *Scinax*, *Leptodactylus*, *Osteopilus*, and *Hypsiboas*, which all rendered relatively low bootstrap values. Also, in the Cophomantinae subfamily, the COI barcode generated contradictory clusters, such as *Bokermannohyla alvarengai* being sister group of *Hypsiboas albopunctatus*, *Dendropsophus minutus* and *Hypsiboas multifasciatus*, and *Aplastodiscus callipygius* and *Dendropsophus cachimbo*, and *Aplastodiscus albosignatus*.

### Intra- and interspecific divergence

All but five of the species collected in the present study were characterized by intraspecific divergence equal to or lower than 9.9% (Suppl. material 1, Table 1). However, higher values were recorded for some taxa, such as *Ranitomeya ventrimaculata* (12.9%), *Hypsiboas leptolineatus*, *Leptodactylus knudseni* (13.3%), and *Scinax fuscomarginatus* with 10.9% (Suppl. material 1).

**Table 1.** Range of intraspecific and between-taxon divergence values recorded in the present study.

Comparison	Percentage distance
Intraespecific (including GenBank sequences): *Atelopus varius*	0.5–1.2
*Craugastor podiciferus*	4.1–11.4
*Dendropsophus labialis*	0.2–9.0
*Engystomops pustulosus*	0.0–11.4
Intraspecific (only species collected during the present study)	0.0–9.9*
Interspecific	11.0–39.0
Between genera	15.0–31.4
Between families (Bufonidae, Dendrobatidae, Hylidae, Craugastoridae, Leiuperidae, Microhylidae, Aromobatidae, and Leptodactylidae)	23.0–31.0

* Excluding the five outliers (see text).

Interspecific divergence varied considerably (Table 1). The distances between most species (5826 comparisons) were within the 9.9-39% range, whereas a few species (60 comparisons) were in the 0-9.9% range. The distances between populations of *Atelopus varius*, *Craugastor podiciferus*, *Dendropsophus labialis*, and *Engystomops pustulosus* exceed the observed intraspecific distances in other species.

## Discussion

A single mitochondrial DNA barcode, derived from the COI gene, identified correctly 93 of the 109 Neotropical amphibian species analyzed in the present study. Similar barcodes (sequences) were not observed in different species, and lower distances (generally 0.0–9.9%) were observed within species than between them. The ranges of values recorded in the present study were consistent with those recorded in previous amphibian studies (Table 2). However, relatively high intraspecific variation was recorded between populations in some species, such as *Engystomops pustulosus* (0.0–11.4%), *Craugastor podiciferus* (4.1–11.4%), and *Dendropsophus labialis* (0.2–9.0%). This indicates the possible presence of additional cryptic species, and supports the development of a standard screening threshold of sequence differentiation that would contribute to the more systematic and effective identification of new animal species.

**Table 2.** Within- and between-taxon distances recorded in different groups of amphibians, based on COI sequences.

Taxon or group	Geographic region	Percentage divergence in the COI gene (mean divergence)		Reference
		Within species	Between species	
Mantellidae (frogs)	Madagascar	10.0–18.0 (5.4)	(20.7)	Vences et al. (2005a)
*Aneides* (Climbing salamanders)	USA	> 7.8 (4.3)	(13.5)	Vences et al. (2005a)
*Litoria fallax* (two lineages)	Australia	5.0	11–12	James and Moritz (2000)
*Ambystoma laterale-jeffesonium* complex	Canada	–	9–14	Smith et al. (2007)
*Scinax ruber*	French Guiana	1.3–14.3	–	Fouquet et al. (2007)
Rhinella gr. margaritifera	Brazil, Ecuador, Peru, French Guiana	1.0–5.1	–	Fouquet et al. (2007)
*Engystomops pustulosus*	Mexico, Guatemala, Nicaragua, Costa Rica, Panama, Colombia, Venezuela	0.0–11.4	–	Data from Weigt et al. (2005) and analyzed in this study
*Dendropsophus minutus*	French Guiana, Suriname, Guyana	–	9 (uncorrected*p*)	Hawkins et al. (2007)
Hynobiidae (Asian salamanders)	China, Korea, Russia, Iran, Afghanistan, Kazakhstan	0.0–0.061	0.007–0.165	Xia et al. (2012)
Chinese amphibians	China	0–0.101	0.031–0.282	Che et al. (2012)
Eight frog families	Bolivia, Brazil, Colombia, Costa Rica, Ecuador, El Salvador, French Guiana, Guatemala, Guyana Haiti, Jamaica, Mexico, Nicaragua, Panama, Peru, Suriname Trinidad and Tobago, USA, Venezuela	0–9.9	11–39	Present study

Lower intra- and interspecific distances have been recorded for the COI barcode in most other animal groups. In butterflies, for example, mean intraspecific distances were 0.46%, while those between species ranged from 2.97% (Hebert et al. 2004b) to 4.58% (Hajibabaei et al. 2006a). In birds, these distances were 0.43% and 7.93%, respectively (Hebert et al. 2004a), in primates, 0.30% and 5.88% (Hajibabaei et al. 2006b), and in fishes, 0.39% and 9.93% (Ward et al. 2005).

The high COI divergence rates recorded in the present study were nevertheless similar to those recorded in pulmonate snails (Thomaz et al. 1996) and lizards (Harris et al. 2004). In order to evaluate the relative divergence of this gene, Vences et al. (2005a) compared substitution rates in COI with those of two other mitochondrial genes commonly used in studies of amphibians (Cytb and ND4), and concluded that molecular evolution in COI is relatively fast, resulting in considerable variability in comparison with either of the other two genes.

The neighbor-joining tree indicated that most of the species and genera analyzed in the present study form relatively cohesive units. However, the data available on *Dendropsophus minutus* (Hawkins et al. 2007; present study) indicate that this form may include more than one species, and a similarly complex situation was observed in the *Atelopus* species (Lotters et al. 2011). The greatest intraspecific distances were recorded in *Ranitomeya ventrimaculata* (12.9%), *Leptodactylus knudseni*, *Hypsiboas leptolineatus* (13.3%), and *Scinax fuscomarginatus* (10.9%). A similar degree of divergence was found in *Ranitomeya ventrimaculata* by Symula et al. (2003) and Brown et al. (2011). Likewise, Kok and Kalamandeen (2008) have suggested that *Leptodactylus knudseni* may represent a species complex. The status of *Hypsiboas leptolineatus* and *Scinax fuscomarginatus* is less clear, especially given the taxonomic complexity of *Scinax*, given the large number of known species, its conservative morphology, and the number of undescribed species (Nunes et al. 2012; Duellman et al. 2016).

The greatest intrageneric distances were recorded in *Hypsiboas* (18.2%), *Craugastor* (19.7%), and *Osteopilus* (20.2%). The considerable distances between some *Craugastor* species indicates the existence of a species complex, as indicated previously for *Craugastor podiciferus* by Streicher et al. (2009). In respect to *Osteopilus septentrionalis*, which is widely distributed in Cuba, a similar pattern was observed by the Cyt b gene (Heinicke et al. 2011). According to theses authors, this may be related to ancient marine incursions, which would have isolated different lineages.

The general polytomy observed in the present study may have been the result of the phylogenetic divergence at the family and genus levels, and the relatively reduced number of terminal taxa. This may also be reflected in the considerable variation in the bootstrap values, from 0% to 92%, found in some clades.

The amplification of the COI gene is straightforward in most vertebrates (Clare et al. 2007; Hajibabaei et al. 2006b; Hebert et al. 2004b; Ward et al. 2005). In the present study, however, difficulties were encountered due to the use of universal primers, as reported previously by Vences et al. (2005a; 2012). For instance, in such studies, several modifications were done to perform successful COI amplifications, such as PCR purification and cloning, annealing temperature optimizations, and others. Thus, it may be necessary to formulate a cocktail of primers, with differentiated amplification protocols and annealing temperatures appropriate to the different amphibian species groups, genera or families (Clare et al. 2007; Vences et al. 2012). However, for other groups, such as Asian Salamanders, Xia et al. (2012) concluded that the high success rate in the sequencing (89%) was due to the reduced variation in the priming regions.

The results of the present study support the use of COI sequences as a DNA barcode for help the identification of Neotropical amphibian species, in particular to ensure the presence of cryptic forms. However, it will still be necessary to identify the factors determining the relatively high rates of divergence observed within the populations of some of the species analyzed in the present study. It will also be important to compile a database of sequences for different molecular markers, in order to better evaluate intra- and inter-specific patterns of variability (Richardson 2012; Luquet et al. 2015; Chambers and Hebert, 2016), addition to update the identification of specimens in the collections.

## Supplementary materials

Supplementary material 1 Data on the specimens examined in the present study Data type: The specimens used in this study, intraspecific distances, locality data and the GenBank association number of the submitted COI sequences. The museum number and reference of each specimen. Explanation note: Please note that some of the sequences used in the study are incompletely referenced in the GenBank barcode database because they lack some data and we are unable to rectify this because the samples were collected too long ago (1980s or before) for the missing data to be found. This dataset is made available under the Open Database License (http://opendatacommons.org/licenses/odbl/1.0/). The Open Database License (ODbL) is a license agreement intended to allow users to freely share, modify, and use this Dataset while maintaining this same freedom for others, provided that the original source and author(s) are credited.Ruth A. Estupiñán, Stephen F. Ferrari, Evonnildo C. Gonçalves, Maria Silvanira R. Barbosa, Marcelo Vallinoto, Maria Paula C. Schneider

## References

[B1] Biju SD Garg S Mahony S Wijayathilaka N Senevirathne G Meegaskumbura M (2014) DNA barcoding, phylogeny and systematics of Golden-backed frogs (*Hylarana*, Ranidae) of the Western Ghats-Sri Lanka biodiversity hotspot, with the description of seven new species. Contributions to Zoology 83: 269–335. http://www.ctoz.nl/cgi/t/text/get-pdf?c=ctz;idno=8304a04

[B2] Blotto BL Nuñez JJ Basso NG Úbeda CA Wheeler WC Faivovich J (2012) Phylogenetic relationships of a Patagonian frog radiation, the Alsodes + Eupsophus clade (Anura: Alsodidae), with comments on the supposed paraphyly of Eupsophus. Cladistics 29: 113–131. doi: 10.1111/j.1096-0031.2012.00417.x 10.1111/j.1096-0031.2012.00417.x 34814377

[B3] Bossuyt F Meegaskumbura M Beenaerts N Gower DJ Pethiyagoda R Roelants K Mannaert A Wilkinson M Bahir MM Manamendra-Arachchi K Ng PKL Schneider CJ Oommen OV Milinkovitch MC (2004) Local endemism within the western Ghats – Sri Lanka biodiversity hotspot. Science 306: 479–481. doi: 10.1126/science.1100167 15486298 10.1126/science.1100167

[B4] Brasil (1979) Lei 6638 de 08 de maio de 1979 Normas para a Prática Didático-científica da Vivissecção de Animais. http://www2.camara.leg.br/legin/fed/lei/1970-1979/lei-6638-8-maio-1979-366514-publicacaooriginal-1-pl.html

[B5] Brown JL Twomey E Amezquita A de Souza MB Caldwell JP Lotters S von May R Melo-Sampaio PR Mejia-Vargas D Perez-Pena P Pepper M Poelman EH Sanchez-Rodriguez M Summers K (2011) A taxonomic revision of the Neotropical poison frog genus *Ranitomeya* (Amphibia: Dendrobatidae). Zootaxa 3083: 1–120. http://www.mapress.com/zootaxa/2011/2/zt03083p120.pdf

[B6] Brown WM George M Jr Wilson AC (1979) Rapid evolution of animal mitochondrial DNA. Proceedings of the National Academy of Sciences of the United States of America 76: 1967–1971. doi: 10.1073/pnas.76.4.1967 109836 10.1073/pnas.76.4.1967 PMC383514

[B7] Chambers EA Hebert PDN (2016) Assessing DNA Barcodes for Species Identification in North American Reptiles and Amphibians in Natural History Collections. PLoS ONE 11: e0154363. doi: 10.1371/journal.pone.0154363 27116180 10.1371/journal.pone.0154363 PMC4846166

[B8] Channing A Schmitz A Burger M Kielgast J (2013) A molecular phylogeny of African Dainty Frogs, with the description of four new species (Anura: Pyxicephalidae: Cacosternum). Zootaxa 3701: 518–550. doi: 10.11646/zootaxa.3701.5.2 26191601 10.11646/zootaxa.3701.5.2

[B9] Che J Chen HM Yang J-X Jin J-Q Jiang K Yuan Z-Y Murphy RW Zhang Y (2012) Universal COI primers for DNA barcoding amphibians. Molecular Ecology Resources 12: 247–258. doi: 10.1111/j.1755-0998.2011.03090 22145866 10.1111/j.1755-0998.2011.03090.x

[B10] Clare EL Lim BK Engstrom MD Eger JL Hebert PDN (2007) DNA barcoding of Neotropical bats: species identification and discovery within Guyana. Molecular Ecology Notes 7: 184–190. doi: 10.1111/j.1471-8286.2006.01657

[B11] Collins JP (2010) Amphibian decline and extinction: what we know and what we need to learn. Diseases of Aquatic Organisms 92: 93–99. doi: 10.3354/dao02307 21268970 10.3354/dao02307

[B12] Crawford AJ Lips KR Bermingham E (2010) Epidemic disease decimates amphibian abundance, species diversity, and evolutionary history in the highlands of central Panama. Proceedings of the National Academy of Sciences of the United States of America 107: 13777–13782. doi: 10.1073/pnas.0914115107 20643927 10.1073/pnas.0914115107 PMC2922291

[B13] Crawford AJ Cruz C Griffith E Ross H Ibañez R Lips KR Driskell AC Bermingham E Crump P (2013) Dna barcoding applied to ex situ tropical amphibian conservation programme reveals cryptic diversity in captive populations. Molecular Ecology Resources 13: 1005–1018. doi: 10.1111/1755-0998.12054 23280343 10.1111/1755-0998.12054

[B14] Darst CR Cannatella DC (2004) Novel relationships among hyloid frogs inferred from 12S and 16S mitochondrial DNA sequences. Molecular Phylogenetics and Evolution 31: 462–475. doi: 10.1016/j.ympev.2003.09.003 15062788 10.1016/j.ympev.2003.09.003

[B15] De la Riva I Köhler J Lötters S Reichle S (2000) Ten years of research on Bolivian amphibians: updated checklist, distribution, taxonomic problems, literature and iconography. Revista Española de Herpetologia 14: 19–164. https://www.researchgate.net/profile/Stefan_Loetters/publication/258507718_Ten_years_of_research_on_Bolivian_amphibians_updated_checklist_distribution_taxonomic_problems_literature_and_iconography/links/0046352861ec6d136d000000.pdf

[B16] Duellman WE Marion AB Hedges SB (2016) Phylogenetics, classification, and biogeography of the treefrogs (Amphibia: Anura: Arboranae). Zootaxa 4104: 1–109. doi: 10.11646/zootaxa.4104.1.1 27394762 10.11646/zootaxa.4104.1.1

[B17] Faivovich J Haddad CFB García PCA Frost DR Campbell JA Wheeler WC (2005) Systematic review of the frog family hylidae with special reference to Hylinae: phylogenetic analysis and taxonomic revision. Bulletin of the American Museum of Natural History 294: 1–240. doi: 10.1206/0003-0090(2005)294[0001:SROTFF]2.0.CO;2

[B18] Ferreira LV Sá RL Buschbacher R Batmanian G Silva JMC Arruda MB Moretti E Sá LFSN Falconer J Bampi MI (2001) Identificação de áreas prioritárias para a conservação da biodiversidade por meio da representatividade das unidades de conservação e tipos de vegetação nas ecorregiões da Amazônia brasileira. In: Capobianco JPR Veríssimo A Moreira A Sawyer D Santos I Pinto LP (Eds) Biodiversidade na Amazônia Brasileira: Avaliação e Ações Prioritárias, Uso Sustentável e Repartição de Benefícios. Instituto Socioambiental, São Paulo, 268–289.

[B19] Fouquet A Gilles A Vences M Marty C Blanc M Gemmell N (2007) Underestimation of species richness in Neotropical frogs revealed by mtDNA analyses. PLoS ONE 2: e1109. doi: 10.1371/journal.pone.0001109 17971872 10.1371/journal.pone.0001109 PMC2040503

[B20] Frost DR (2016) Amphibian Species of the World: an Online Reference. Version 6.0 (05/05/2014 access). Electronic Database accessible at http://research.amnh.org/herpetology/amphibia/index.html. American Museum of Natural History, New York, USA.

[B21] Frost DR Grant T Faivovich J Bain RH Haas A Haddad CFB de Sa RO Channing A Wilkinson M Donnellan SC Raxworthy CJ Campbell JA Blotto BL Moler P Drewes RC Nussbaum RA Lynch JD Green DM Wheeler WC (2010) The Amphibian tree of life. Bulletin of the American Museum of Natural History 297: 1–370. doi: 10.1206/0003-0090(2006)297[0001:TATOL]2.0.CO;2

[B22] Funk WC Caminer M Ron SR (2012) High levels of cryptic species diversity uncovered in Amazonian frogs. Proceedings of the Royal Society B: Biological Sciences 279: 1806–1814. doi: 10.1098/rspb.2011.1653 22130600 10.1098/rspb.2011.1653 PMC3297442

[B23] Grant T Frost DR Caldwell JP Gagliardo R Haddad CFB Kok PJR Means DB Noonan BP Schargel WE Wheeler WC (2006) Phylogenetic systematics of dart-poison frogs and their relatives (Amphibia: Athesphatanura: Dendrobatidae). Bulletin of the American Museum of Natural History 299: 1–262. doi: 10.1206/0003-0090(2006)299[1:PSODFA]2.0.CO;2

[B24] Hajibabaei M Janzen DH Burns JM Hallwachs W Hebert PDN (2006a) DNA barcodes distinguish species of tropical Lepidoptera. Proceedings of the National Academy of Sciences of the United States of America 103: 968–971. doi: 10.1073/pnas.0510466103 16418261 10.1073/pnas.0510466103 PMC1327734

[B25] Hajibabaei M Singer GAC Hickey DA (2006b) Benchmarking DNA barcodes: an assessment using available primate sequences. Genome 49: 851–854. doi: 10.1139/G06-025 16936793 10.1139/g06-025

[B26] Hall TA (1999) BioEdit: a user-friendly biological sequence alignment editor and analysis program for Windows 95/98/NT. Nucleic Acids Symposium Series 41: 95–98. http://brownlab.mbio.ncsu.edu/JWB/papers/1999Hall1.pdf

[B27] Harris DJ Batista V Lymberakis P Carretero MA (2004) Complex estimates of evolutionary relationships in *Tarentola mauritanica* (Reptilia: Gekkonidae) derived from mitochondrial DNA sequences. Molecular Phylogenetics and Evolution 30: 855–859. doi: 10.1016/S1055-7903(03)00260-4 15012965 10.1016/S1055-7903(03)00260-4

[B28] Hasan M Islam MM Kuramoto M Kurabayashi A Sumida M (2014) Description of two new species of Microhyla (Anura: Microhylidae) from Bangladesh. Zootaxa 3755: 401–418. doi: 10.11646/zootaxa.3755.5.1 24869829 10.11646/zootaxa.3755.5.1

[B29] Hawkins MA Sites JW Noonan BP (2007) *Dendropsophus minutus* (Anura: Hylidae) of the Guiana Shield: using DNA barcodes to assess identity and diversity. Zootaxa 1540: 61–67. http://bnoonan.org/Papers/Hawkinsetal_07.pdf

[B30] Hebert PDN Ratnasingham S de Waard JR (2003) Barcoding animal life: Cytochrome c oxidase subunit 1 divergences among closely related species. Proceedings of the Royal Society B: Biological Sciences 270: S596–S599. doi: 10.1098/rsbl.2003.0025 10.1098/rsbl.2003.0025 PMC1698023 12952648

[B31] Hebert PDN Stoeckle MY Zemlak TS Francis CM (2004a) Identification of birds through DNA barcodes. PLoS Biology 2: e312. doi: 10.1371/journal.pbio.0020312 15455034 10.1371/journal.pbio.0020312 PMC518999

[B32] Hebert PDN Penton EH Burns JM Janzen DH Hallwachs W (2004b) Ten species in one: DNA barcoding reveals cryptic species in the Neotropical skipper butterfly *Astraptes fulgerator*. Proceedings of the National Academy of Sciences of the United States of America 101: 14812–14817. doi: 10.1073/pnas.0406166101 15465915 10.1073/pnas.0406166101 PMC522015

[B33] Heinicke M Diaz LM Hedges SB (2011) Origin of invasive Florida frogs traced to Cuba. Biology Letters 7: 407–410. doi: 10.1098/rsbl.2010.1131 21270024 10.1098/rsbl.2010.1131 PMC3097879

[B34] Hoffmann M Hilton-Taylor C Angulo A et al. (2010) The impact of conservation on the status of the World’s vertebrates. Science 330: 1503–1509. doi: 10.1126/science.1194442 20978281 10.1126/science.1194442

[B35] James CH Moritz C (2000) Intraspecific phylogeography in the sedge frog *Litoria fallax* (Hylidae) indicates pre-Pleistocence vicariance of an open forest species from eastern Australia. Moleular Ecology 9: 349–358. doi: 10.1046/j.1365-294x.2000.00885.x 10.1046/j.1365-294x.2000.00885.x 10736032

[B36] Kimura M (1980) A simple method for estimating evolutionary rates of base substitutions through comparative studies of nucleotide sequences. Journal of Molecular Evolution 16: 111–120. http://eclass.uoa.gr/modules/document/file.php/D473/%CE%92%CE%B9%CE%B2%CE%BB%CE%B9%CE%BF%CE%B3%CF%81%CE%B1%CF%86%CE%AF%CE%B1/Phylogeny/Kimura_1980.pdf 7463489 10.1007/BF01731581

[B37] Kok PJR Kalamandeen M (2008) Introduction to the taxonomy of the amphibians of Kaieteur National Park Guyana. Abc Taxa. 5: 1–288. http://www.abctaxa.be/downloads/volume-5-introduction-taxonomy-amphibians

[B38] Larson A Chippindale P (1993) Molecular approaches to the evolutionary biology of plethodontid salamanders. Herpetologica. 49: 204–215. http://www.jstor.org/stable/3892797

[B39] Lötters S van der Meijden A Coloma LA Boistel R Cloetens P Ernst R Lehr E Veith M (2011) Assessing the molecular phylogeny of a near extinct. Systematics and Biodiversity 9: 45–57. doi: 10.1080/14772000.2011.557403

[B40] Luquet E Léna JP Miaud C Plénet S (2015) Phenotypic divergence of the common toad (*Bufo bufo*) along an altitudinal gradient: evidence for local adaptation. Heredity 114: 69–79. doi: 10.1038/hdy.2014.71 25074572 10.1038/hdy.2014.71 PMC4815602

[B41] McCallum ML (2007) Amphibian decline or extinction? Current declines dwarf background extinction rate. Journal of Herpetology 41: 483–491. doi: 10.1670/0022-1511(2007)41[483:ADOECD]2.0.CO;2

[B42] Mindell DP Sorenson MD Huddleston CJ Miranda HC Jr Knight A et al. (1997) Phylogenetic relationships among and within select avian orders based on mitochondrial DNA. In: Mindell DP (Ed.) Avian molecular evolution and systematics. Academic Press, New York, 214–247. doi: 10.1016/b978-012498315-1/50014-5

[B43] Moore WS (1995) Inferring phylogenies from mtDNA variation: Mitochondrial-gene trees versus nuclear-gene trees. Evolution 49: 718–726. doi: 10.2307/2410325 10.1111/j.1558-5646.1995.tb02308.x 28565131

[B44] Narins PM Meenderink SWF (2014) Climate change and frog calls: long-term correlations along a tropical altitudinal gradient. Proceedings of the Royal Society B: Biological Sciences 281: 1471–2954. doi: 10.1098/rspb.2014.0401 10.1098/rspb.2014.0401 PMC3996621 24718765

[B45] Nascimento FP Avila-Pires TCS Cunha OR (1988) Répteis Squamata de Rondônia e Mato Grosso coletados através do Programa Polonoroeste. Boletim Museu Paraense Emílio Goeldi série Zoologia 4: 21–66.

[B46] Newman CE Feinberg JA Rissler LJ Burger J Shaffer HB (2012) A new species of leopard frog (Anura: Ranidae) from the urban northeastern US. Molecular Phylogenetics and Evolution 63: 445–455. doi: 10.1016/j.ympev.2012.01.021 22321689 10.1016/j.ympev.2012.01.021 PMC4135705

[B47] Nunes I Kwet A Pombal JP Jr (2012) Taxonomic revision of the Scinax alter species complex (Anura: Hylidae). Copeia 2012: 554–569. doi: 10.1643/CH-11-088

[B48] Pereira JL Lisboa LS Thales MC Lima AS Ferrari SF (2004) Mapa da cobertura da terra na região do alto rio dos Marmelos. In: Anais do I Simpósio de Ciências Geodésicas e Tecnologias da Geoinformação. I Simpósio de Ciências Geodésicas e Tecnologias da Geoinformação Recife (Brazil) CD-Rom http://docplayer.com.br/5332509-Mapa-da-cobertura-da-terra-na-regiao-do-alto-rio-dos-marmelos.html

[B49] Pereyra MO Baldo D Blotto BL Iglesias PP Thomé MTC Haddad CFB Barrio-Amorós C Ibañez R Faivovich J (2016) Phylogenetic relationships of toads of the Rhinella granulosa group (Anura: Bufonidae): A molecular perspective with comments on hybridization and introgression. Cladistics 32: 36–53. doi: 10.1111/cla.12110 10.1111/cla.12110 34732018

[B50] Palumbi SR (1996) Nucleic acids II: the polymerase chain reaction. In: Hillis DM Moritz C Mable BK (Eds) Molecular Systematics. Sinauer Associates, Sunderland, MA, 205–247.

[B51] Pronabio (2002) Programa Nacional da Diversidade Biológica. Ministério do Meio Ambiente Avaliação e identificação de ações prioritárias para a conservação utilização sustentável e repartição dos benefícios da biodiversidade na Amazônia brasileira. MMA/SBF Brasília.

[B52] Real R Barbosa AM Solano IM García-París M (2005) Distinguishing the distributions of two cryptic frogs (Anura: Discoglossidae) using molecular data and environmental modeling. Canadian Journal of Zoology 83: 536–545. doi: 10.1139/z05-040

[B53] Richardson JL (2012) Divergent landscape effects on population connectivity in two co-occurring amphibian species. Molecular Ecology 21: 4437–4451. doi: 10.1111/j.1365-294X.2012.05708.x 22891686 10.1111/j.1365-294X.2012.05708.x

[B54] Roelants K Gower DJ Wilkinson M Loader SP Biju SD Guillaume K Moriau L Bossuyt F (2007) Global patterns of diversification in the history of modern amphibians. Proceedings of the National Academy of Sciences of the United States of America 104: 887–892. doi: 10.1073/pnas.0608378104 17213318 10.1073/pnas.0608378104 PMC1783409

[B55] Rowley J Brown R Bain R Kusrini M Inger R Stuart B Wogan G Thy N Chan-ard T Trung CT Diesmos A Iskandar DT Lau M Ming LT Makchai S Truong NQ Phimmachak S (2010) Impending conservation crisis for Southeast Asian amphibians. Biology Letters 6: 336–338. doi: 10.1098/rsbl.2009.0793 20007165 10.1098/rsbl.2009.0793 PMC2880038

[B56] Sambroo KJ Rusell DW (2001) Molecular cloning-laboratory manuals. Cold Spring Harbor, New York, 999 pp.

[B57] Smith MA Poyarkov NA Jr Hebert PDN (2008) COI DNA barcoding amphibians: take the chance. meet the challenge. Molecular Ecology Resources 8: 235–246. doi: 10.1111/j.1471-8286.2007.01964.x 21585765 10.1111/j.1471-8286.2007.01964.x

[B58] Stotz DF Lanyon SM Schulenberg TS Willard DE Peterson T Fitzpatrick J (1997) Avifauna Survey of two tropical forest localities on the middle Rio Jiparaná Rondônia Brazil. Ornithological Monographs 48: 763–781. doi: 10.2307/40157566

[B59] Streicher JW Crawford AJ Edwards CW (2009) Multilocus molecular phylogenetic analysis of the montane *Craugastor podiciferus* species complex (Anura: Craugastoridae) in Isthmian Central America. Molecular Phylogenetic and Evolution 53: 620–630. doi: 10.1016/j.ympev.2009.07.011 10.1016/j.ympev.2009.07.011 19602442

[B60] Stuart SN Chanson JS Cox NA Young BE Rodrigues ASL Fischman DL Waller RW (2004) Status and trends of amphibian declines and Extinctions worldwide. Science 306: 1783–1786. doi: 10.1126/science.1103538 15486254 10.1126/science.1103538

[B61] Stuart B Inger R Voris H (2006) High level of cryptic species diversity revealed by sympatric lineages of Southeast Asian forest frogs. Biological Letters 2: 470–474. doi: 10.1098/rsbl.2006.0505 10.1098/rsbl.2006.0505 PMC1686201 17148433

[B62] Symula R Schulte R Summers K (2003) Molecular systematics and phylogeography of Amazonian poison frogs of the genus *Dendrobates*. Molecular Phylogenetics and Evolution 26: 452–475. doi: 10.1016/S1055-7903(02)00367-6 12644404 10.1016/s1055-7903(02)00367-6

[B63] Tamura K Peterson D Peterson N Stecher G Nei M Kumar S (2011) MEGA5: Molecular evolutionary genetics analysis using maximum likelihood evolutionary distance and maximum parsimony methods. Molecular Biological Evolution 28: 2731–2739. doi: 10.1093/molbev/msr121 10.1093/molbev/msr121 PMC3203626 21546353

[B64] Thomaz D Guiller A Clarke B (1996) Extreme divergence of mitochondrial DNA within species of pulmonate land snails. Proceedings of the Royal Society B: Biological Sciences 263: 363–368. doi: 10.1098/rpb.1996.0056 8920257 10.1098/rspb.1996.0056

[B65] Vences M Kosuch J Boistel R Haddad CFB La Marca E Lötters S Veith M (2003) Convergent evolution of aposematic coloration in Neotropical poison frogs: a molecular phylogenetic perspective. Organisms Diversity and Evolution 3: 215–226. doi: 10.1078/1439-6092-00076

[B66] Vences M Thomas M Bonett RM Vieites DR (2005a) Deciphering amphibian diversity through DNA barcoding: chances and challenges. Philosophical transactions of the Royal Society of London, Series B Biological Sciences 360: 1859–1868. doi: 10.1098/rstb.2005.1717 16221604 10.1098/rstb.2005.1717 PMC1609216

[B67] Vences M Thomas M Van Der Meijden A Chiari Y Vieites D (2005b) Comparative performance of the 16S rRNA gene in DNA barcoding of amphibians. Frontiers in Zoology 2: 1–12. doi: 10.1186/1742-9994-2-5 15771783 10.1186/1742-9994-2-5 PMC555853

[B68] Vences M Nagy ZT Sonet G Verheyen E (2012) DNA barcoding amphibians and reptiles In DNA Barcodes: Methods and Protocols. In: Kress WJ Erickson DL (Eds) Springer Protocols Methods in Molecular Biology 858: 79–107. doi: 10.1007/978-1-61779-591-6_5 10.1007/978-1-61779-591-6_5 22684953

[B69] Vieites DR Wollenberg KC Andreone F Kohler J Glaw F Vences M (2009) Vast underestimation of Madagascar’s biodiversity evidenced by an integrative amphibian inventory. Proceedings of the National Academy of Sciences of the United States of America 106: 8267–8272. doi: 10.1073/pnas.0810821106 19416818 10.1073/pnas.0810821106 PMC2688882

[B70] Ward RD Zemlak TS Innes BH Last PR Hebert PDN (2005) DNA barcoding Australia’s fish species. Philosophical transactions of the Royal Society of London, Series B Biological Sciences 360: 1847–1857. doi: 10.1098/rstb.2005.1716 16214743 10.1098/rstb.2005.1716 PMC1609232

[B71] Weigt LA Crawford AJ Rand AS Ryan M (2005) Biogeography of the túngara frog. *Physalaemus pustulosus*: molecular perspective. Molecular Ecology 14: 3857–3876. doi: 10.1111/j.1365-294X.2005.02707.x 16202101 10.1111/j.1365-294X.2005.02707.x

[B72] Xia Y Gu HF Peng R Chen Q Zheng YC Murphy RW Zeng XM (2012) COI is better than 16s rRNA for DNA barcoding Asiatic Salamanders (Amphibia: Caudata: Hynobiidae). Molecular Ecology Resources 12: 48–56. doi: 10.1111/j.1755-0998.2011.03055.x 21824335 10.1111/j.1755-0998.2011.03055.x

[B73] Xia X (2013) Dambe5: A comprehensive software package for data analysis in molecular biology and evolution. Molecular Biology and Evolution 30: 1720–1728. doi: 10.1111/j.1755-0998.2011.03055.x 23564938 10.1093/molbev/mst064 PMC3684854

